# Supplementary material for: Integrating digital health technologies into the healthcare system: Challenges and opportunities in Nigeria
Source: PLOS Digit Health. 2025 Jul 24;4(7):e0000928. doi: 10.1371/journal.pdig.0000928 (PMC12289021; doi:10.1371/journal.pdig.0000928)
Supplement: S2 Appendix — (DOCX) [file pdig.0000928.s002.docx]

**S2 Appendix: Search Strategy for Different Data Bases**

**PubMed Search Strategy**

| **S/N** | **Keywords** | **Search Strategy** | **Results** |
| --- | --- | --- | --- |
| #6 |  | Search: #1 AND #2 AND #3 Filters: from 2014/7/1 - 2024/6/30 | [919](https://pubmed.ncbi.nlm.nih.gov/?term=%231+AND+%232+AND+%234&filter=dates.2014%2F7%2F1-2024%2F6%2F30&ac=no&sort=relevance) |
| #5 |  | Search: #1 AND #2 AND #3 | [1,05](https://pubmed.ncbi.nlm.nih.gov/?term=%231+AND+%232+AND+%234&ac=no&sort=relevance)4 |
| #4 | Integration | Search: (((((((Integration[Title/Abstract]) OR (implementation[Title/Abstract])) OR (adoption[Title/Abstract])) OR (scalability[Title/Abstract])) OR (sustainability[Title/Abstract])) OR (cost-effectiveness[Title/Abstract])) OR (policy[Title/Abstract])) OR (regulation[Title/Abstract]) | [2,089,953](https://pubmed.ncbi.nlm.nih.gov/?term=%28%28%28%28%28%28%28Integration%5BTitle%2FAbstract%5D%29+OR+%28implementation%5BTitle%2FAbstract%5D%29%29+OR+%28adoption%5BTitle%2FAbstract%5D%29%29+OR+%28scalability%5BTitle%2FAbstract%5D%29%29+OR+%28sustainability%5BTitle%2FAbstract%5D%29%29+OR+%28cost-effectiveness%5BTitle%2FAbstract%5D%29%29+OR+%28policy%5BTitle%2FAbstract%5D%29%29+OR+%28regulation%5BTitle%2FAbstract%5D%29&ac=no&sort=relevance) |
| #3 | Sub-Saharan Africa | Search: (((((((((((((((((((((((((((((((((((((((((((((((((((((((((sub-Saharan Africa[Title/Abstract]) OR (sub Saharan Africa[Title/Abstract])) OR (Subsaharan Africa[Title/Abstract])) OR (developing countries[Title/Abstract])) OR (low-and middle-income countries[Title/Abstract])) OR (LMICs[Title/Abstract])) OR (Angola[Title/Abstract])) OR (Benin[Title/Abstract])) OR (Botswana[Title/Abstract])) OR (Burkina Faso[Title/Abstract])) OR (Burundi[Title/Abstract])) OR (Cape Verde[Title/Abstract])) OR (Cameroon[Title/Abstract])) OR (Central Africa Republic[Title/Abstract])) OR (Chad[Title/Abstract])) OR (Comoros[Title/Abstract])) OR (Congo[Title/Abstract])) OR (Democratic Republic of the Congo[Title/Abstract])) OR (Djibouti[Title/Abstract])) OR (Equatorial Guinea[Title/Abstract])) OR (Eritrea[Title/Abstract])) OR (Eswatini[Title/Abstract])) OR (Ethiopia[Title/Abstract])) OR (Gabon[Title/Abstract])) OR (Gambia[Title/Abstract])) OR (Ghana[Title/Abstract])) OR (Guinea[Title/Abstract])) OR (Guinea-Bissau[Title/Abstract])) OR (ivory coast[Title/Abstract])) OR (Cote d'Ivoire[Title/Abstract])) OR (Kenya[Title/Abstract])) OR (Lesotho[Title/Abstract])) OR (Liberia[Title/Abstract])) OR (Madagascar[Title/Abstract])) OR (Malawi[Title/Abstract])) OR (Mali[Title/Abstract])) OR (Mauritania[Title/Abstract])) OR (Mauritius[Title/Abstract])) OR (Mozambique[Title/Abstract])) OR (Namibia[Title/Abstract])) OR (Niger[Title/Abstract])) OR (Nigeria[Title/Abstract])) OR (Rwanda[Title/Abstract])))) OR (Sao Tome and Principe[Title/Abstract])) OR (Senegal[Title/Abstract])) OR (Seychelles[Title/Abstract])) OR (Sierra Leone[Title/Abstract])) OR (Somalia[Title/Abstract])) OR (South Africa[Title/Abstract])) OR (South Sudan[Title/Abstract])) OR (Sudan[Title/Abstract])) OR (Tanzania[Title/Abstract])) OR (Togo[Title/Abstract])) OR (Uganda[Title/Abstract])) OR (Zambia[Title/Abstract])) OR (Zimbabwe[Title/Abstract]) | [533,](https://pubmed.ncbi.nlm.nih.gov/?term=longqueryf04a6f20cf1d0fb86da0&ac=no&sort=relevance)367 |
| #2 | Healthcare systems | Search: (((((((healthcare systems[Title/Abstract]) OR (healthcare system[Title/Abstract])) OR (healthcare delivery[Title/Abstract])) OR (health services[Title/Abstract])) OR (healthcare services[Title/Abstract])) OR (public health[Title/Abstract])) OR (health workforce[Title/Abstract])) OR (health equity[Title/Abstract]) | [58](https://pubmed.ncbi.nlm.nih.gov/?term=%28%28%28%28%28%28%28healthcare+systems%5BTitle%2FAbstract%5D%29+OR+%28healthcare+system%5BTitle%2FAbstract%5D%29%29+OR+%28healthcare+delivery%5BTitle%2FAbstract%5D%29%29+OR+%28health+services%5BTitle%2FAbstract%5D%29%29+OR+%28healthcare+services%5BTitle%2FAbstract%5D%29%29+OR+%28public+health%5BTitle%2FAbstract%5D%29%29+OR+%28health+workforce%5BTitle%2FAbstract%5D%29%29+OR+%28health+equity%5BTitle%2FAbstract%5D%29&ac=no&sort=relevance)5,012 |
| #1 | Digital Health | Search: (((((((digital health[Title/Abstract]) OR (digital health technologies[Title/Abstract])) OR (ehealth[MeSH Terms])) OR (mhealth[Title/Abstract])) OR (telemedicine[Title/Abstract])) OR (telehealth[Title/Abstract])) OR (health informatics[Title/Abstract])) OR (health information systems[Title/Abstract]) | [86,](https://pubmed.ncbi.nlm.nih.gov/?term=%28%28%28%28%28%28%28digital+health%5BTitle%2FAbstract%5D%29+OR+%28digital+health+technologies%5BTitle%2FAbstract%5D%29%29+OR+%28ehealth%5BMeSH+Terms%5D%29%29+OR+%28mhealth%5BTitle%2FAbstract%5D%29%29+OR+%28telemedicine%5BTitle%2FAbstract%5D%29%29+OR+%28telehealth%5BTitle%2FAbstract%5D%29%29+OR+%28health+informatics%5BTitle%2FAbstract%5D%29%29+OR+%28health+information+systems%5BTitle%2FAbstract%5D%29&ac=no&sort=relevance)444 |

**Web of Science Search Strategy**

| S/N | KEYWORDS | SEARCH STRATEGY | RESULTS |
| --- | --- | --- | --- |
| #1 | Digital health | **TS=("Digital health technologies" OR ehealth OR mhealth OR telemedicine OR "health informatics" OR "health information system")** and **Preprint Citation Index** (Exclude – Database) | [99,018](http://webofscience-clarivate-cn-s.webvpn.njmu.edu.cn:8118/wos/alldb/summary/bf89d952-12de-4af5-80d0-8f25fcfb1598-0104587ebb/relevance/1) |
| #2 | Healthcare system | **TS=("Healthcare system*" OR "Healthcare delivery" OR "Health services" OR "Healthcare services" OR "Public health" OR "Health workforce" OR "Health equity")** and **Preprint Citation Index** (Exclude – Database) | [3,220,175](http://webofscience-clarivate-cn-s.webvpn.njmu.edu.cn:8118/wos/alldb/summary/f271bc09-ae83-4860-9b4c-2319c3a2d72e-0104588da1/relevance/1) |
| #3 | #1 AND #2 | **#1 AND #2 and Preprint Citation Index (Exclude – Database)** | [27,085](http://webofscience-clarivate-cn-s.webvpn.njmu.edu.cn:8118/wos/alldb/summary/8b1d7a64-f315-43ab-ba49-03d76a8b25c2-0104588fa8/relevance/1) |
| #4 | Filters | #1 AND #2 and Preprint Citation Index (Exclude – Database) and SOUTH AFRICA or KENYA or NIGERIA or ETHIOPIA or MALAYSIA or UGANDA or GHANA or EGYPT or TANZANIA or BURUNDI or SIERRA LEONE or BENIN or ALGERIA or COTE IVOIRE or REP CONGO or DEM REP CONGO or SENEGAL or MALI or BURKINA FASO or MOROCCO or BOTSWANA or ZAMBIA or MOZAMBIQUE or RWANDA or CAMEROON or ZIMBABWE or MALAWI or LIBYA or LIBERIA or MAURITIUS or TOGO or ANGOLA or ESWATINI or GAMBIA or MONGOLIA or NIGER or GABON or DJIBOUTI (Countries/Regions) and 2024 or 2023 or 2022 or 2021 or 2020 or 2019 or 2018 or 2017 or 2016 or 2015 or 2014 (Publication Years) and Web of Science Core Collection (Database) | [1,628](http://webofscience-clarivate-cn-s.webvpn.njmu.edu.cn:8118/wos/alldb/summary/daa5d109-8d97-4a8e-bbf0-09b978efa7b0-010458af01/relevance/1) |

**CINAHL Search Strategy**

| **Search** | **Search terms** | **Search strategy** | **Results** |
| --- | --- | --- | --- |
| S4 |  | S1 AND S2  Limiters - Publication Date: 20140701-20240831; Geographic Subset: Africa | (19) |
| S3 |  | S1 AND S2 | (10,683) |
| S2 | Healthcare system | "Healthcare system*" OR "Healthcare delivery" OR "Health services" OR "Healthcare services" OR "Public health" OR "Health workforce" OR "Health equity" | (510,795) |
| S1 | Digital health | "Digital health technologies" OR ehealth OR mhealth OR telemedicine OR "health informatics" OR "health information system" | (46,843) |

**SCOPUS Search Strategy**

| **Search** | **Search terms** | **Search strategy** | **Results** |
| --- | --- | --- | --- |
| 4 |  | ( TITLE-ABS-KEY ( "Digital health technologies" OR ehealth OR mhealth OR telemedicine OR "health informatics" OR "health information system" ) ) AND ( TITLE-ABS-KEY ( "Healthcare system*" OR "Healthcare delivery" OR "Health services" OR "Healthcare services" OR "Public health" OR "Health workforce" OR "Health equity" ) ) AND PUBYEAR > 2013 AND PUBYEAR < 2025 AND ( LIMIT-TO ( AFFILCOUNTRY , "South Africa" ) OR LIMIT-TO ( AFFILCOUNTRY , "Nigeria" ) OR LIMIT-TO ( AFFILCOUNTRY , "Kenya" ) OR LIMIT-TO ( AFFILCOUNTRY , "Ghana" ) OR LIMIT-TO ( AFFILCOUNTRY , "Uganda" ) OR LIMIT-TO ( AFFILCOUNTRY , "Tanzania" ) OR LIMIT-TO ( AFFILCOUNTRY , "Malawi" ) OR LIMIT-TO ( AFFILCOUNTRY , "Zimbabwe" ) OR LIMIT-TO ( AFFILCOUNTRY , "Mozambique" ) OR LIMIT-TO ( AFFILCOUNTRY , "Rwanda" ) OR LIMIT-TO ( AFFILCOUNTRY , "Namibia" ) OR LIMIT-TO ( AFFILCOUNTRY , "Zambia" ) OR LIMIT-TO ( AFFILCOUNTRY , "Botswana" ) OR LIMIT-TO ( AFFILCOUNTRY , "Cameroon" ) OR LIMIT-TO ( AFFILCOUNTRY , "Congo" ) OR LIMIT-TO ( AFFILCOUNTRY , "Burkina Faso" ) OR LIMIT-TO ( AFFILCOUNTRY , "Senegal" ) OR LIMIT-TO ( AFFILCOUNTRY , "Democratic Republic Congo" ) OR LIMIT-TO ( AFFILCOUNTRY , "Mali" ) OR LIMIT-TO ( AFFILCOUNTRY , "Sierra Leone" ) OR LIMIT-TO ( AFFILCOUNTRY , "Madagascar" ) OR LIMIT-TO ( AFFILCOUNTRY , "Cote d'Ivoire" ) OR LIMIT-TO ( AFFILCOUNTRY , "Lesotho" ) OR LIMIT-TO ( AFFILCOUNTRY , "Swaziland" ) OR LIMIT-TO ( AFFILCOUNTRY , "Somalia" ) OR LIMIT-TO ( AFFILCOUNTRY , "Mauritius" ) OR LIMIT-TO ( AFFILCOUNTRY , "Burundi" ) OR LIMIT-TO ( AFFILCOUNTRY , "Liberia" ) OR LIMIT-TO ( AFFILCOUNTRY , "Benin" ) OR LIMIT-TO ( AFFILCOUNTRY , "Niger" ) OR LIMIT-TO ( AFFILCOUNTRY , "Gabon" ) OR LIMIT-TO ( AFFILCOUNTRY , "Guinea" ) OR LIMIT-TO ( AFFILCOUNTRY , "Togo" ) OR LIMIT-TO ( AFFILCOUNTRY , "Angola" ) OR LIMIT-TO ( AFFILCOUNTRY , "Gambia" ) ) | [1,524 results](https://www.scopus.com/search/history/results.uri?origin=searchhistory&shid=7) |
| 3 |  | ( TITLE-ABS-KEY ( "Digital health technologies" OR ehealth OR mhealth OR telemedicine OR "health informatics" OR "health information system" ) ) AND ( TITLE-ABS-KEY ( "Healthcare system*" OR "Healthcare delivery" OR "Health services" OR "Healthcare services" OR "Public health" OR "Health workforce" OR "Health equity" ) ) | [31,708 results](https://www.scopus.com/search/history/results.uri?origin=searchhistory&shid=4) |
| 2 | **Healthcare system** | TITLE-ABS-KEY ( "Healthcare system*" OR "Healthcare delivery" OR "Health services" OR "Healthcare services" OR "Public health" OR "Health workforce" OR "Health equity" ) | [1,606,874 results](https://www.scopus.com/search/history/results.uri?origin=searchhistory&shid=2) |
| 1 | **Digital health** | TITLE-ABS-KEY ( "Digital health technologies" OR ehealth OR mhealth OR telemedicine OR "health informatics" OR "health information system" ) | [121,909 results](https://www.scopus.com/search/history/results.uri?origin=searchhistory&shid=1) |
