# Supplementary material for: Integrating digital health technologies into the healthcare system: Challenges and opportunities in Nigeria
Source: PLOS Digit Health. 2025 Jul 24;4(7):e0000928. doi: 10.1371/journal.pdig.0000928 (PMC12289021; doi:10.1371/journal.pdig.0000928)
Supplement: S3 Appendix — (DOCX) [file pdig.0000928.s003.docx]

**S3 Appendix: List of Excluded Studies and Reasons for Exclusion**

| **Number Excluded** | **Study ID** | **Title** | **Reason for Exclusion** |
| --- | --- | --- | --- |
| n = 3 | 1. Birdling et al., 2023  2. Akwaowo et al., 2022  3. Ezeonwumelu et al., 2022 | 1. Knowledge and Perception of Telemedicine among Medical Students of  the University of Jos, Plateau State, Nigeria  2. Adoption of electronic medical records in developing countries- A multi-state study of the Nigerian healthcare system  3. Healthcare provider-to patient perspectives on the uptake of teleconsultation services in the Nigerian healthcare system during the COVID-19 pandemic era | No specified digital health intervention |
| n = 4 | 1. Olubiyi et al., 2022  2. Bammeke et al., 2023  3. Olubiyi, 2022  Obeta, 2022 |  | 1. Cross-sectional studies on knowledge and perceptions, no DHT implemented  2. Assessment of open data kit mobile technology adoption to enhance reporting of supportive supervision concucted for oval poliovirus vaccine supplementary immunization activities in Nigeria, March 2017-February 2020  3. Availability and Utilization of Digital Health Technology for  Improved Patients Care: A Cross-Sectional Study of Nurses’  Perspectives at a State General Hospital in North-Central Nigeria |
| n = 1 | Ahonkhai et al., 2021 | PEERNaija: A gamified mHealth behavioural intervention to improve adherence to antiretroviral treatment among adolescents and young adults in Nigeria | Descriptive preliminary study, DHT not yet implemented |
| n = 1 | Eze et al., 2015 | What experts think about integrating mobile health into routine immunization service delivery in Nigeria | No sample size |
| n = 1 | Okunade et al., 2020 | Impact of mobile technologies on cervical cancer screening  practices in Lagos, Nigeria (mHealth-Cervix): Protocol for a  randomised controlled trial | Research protocol |
| n = 1 | Eleje et al., 2022 | Mother-to-child transmission of human  immunodeficiency virus, hepatitis B virus  and hepatitis C virus among pregnant women  with single, dual or triplex infections of  human immunodeficiency virus, hepatitis B  virus and hepatitis C virus in Nigeria:  A systematic review and meta-analysis | Systematic review |
