# Supplementary material for: Integrating digital health technologies into the healthcare system: Challenges and opportunities in Nigeria
Source: PLOS Digit Health. 2025 Jul 24;4(7):e0000928. doi: 10.1371/journal.pdig.0000928 (PMC12289021; doi:10.1371/journal.pdig.0000928)
Supplement: S4 Appendix — (DOCX) [file pdig.0000928.s004.docx]

**S4 Appendix- Summary of the Mixed Method Appraisal Tool for Quality Assessment of Included Studies**

|  | **Methodological quality criteria** | | **Qualitative-Total Yes** | **Quantitative randomized controlled trials-Total Yes** | **Quantitative non-randomized- Total Yes** | **Quantitative descriptive- Total Yes** | **Mixed methods- Total Yes** | **TOTAL SCORE** |
| --- | --- | --- | --- | --- | --- | --- | --- | --- |
| Adewuya et al., | YES | YES | - | 4 |  |  |  | 80% |
| Akamike et al., 2021 | YES | MAYBE |  | 2 |  |  |  | 40% |
| Akande et al. | YES | YES |  | 5 |  |  |  | 100% |
| Akeju et al., | YES | YES |  |  |  |  | 4 | 80% |
| Ayamolowo et al. | YES | YES |  |  |  |  | 5 | 100% |
| Babalola et al., | YES | YES |  | 2 |  |  |  | 40% |
| Birukila et al., | YES | MAYBE |  |  |  | 5 |  | 100% |
| Cremers et al. | YES | YES | 5 |  |  |  |  | 100% |
| Ebenso et al. | YES | YES |  |  |  |  | 5 | 100% |
| Fox et al., 2022 | YES | YES | 5 |  |  |  |  | 100% |
| Hicks et al. | YES | YES |  |  |  |  | 5 | 100% |
| Itanyi et al., 2023 | YES | YES | 5 |  |  |  |  | 100% |
| Kenny et al. | YES | NO | 1 |  |  |  |  | 10% |
| Kuhns et al., 2021 | YES | YES | 5 |  |  |  |  | 100% |
| McNabb et al., 2015 | YES | YES |  |  | 4 |  |  | 80% |
| Musa et al., 2023 | YES | YES | 0 |  |  |  |  | 0% |
| Obi-Jeff et al., 2022 | YES | YES | 5 |  |  |  |  | 100% |
| Odu et al., 2024 | YES | YES |  |  | 3 |  |  | 60% |
| Olajubu et al., 2020 | YES | YES |  | 3 |  |  |  | 60% |
| Olajubu et al., 2022 | YES | YES | 5 |  |  |  |  | 100% |
| Olayiwola et al., 2020 | YES | MAYBE |  |  |  | 1 |  | 10% |
| Onyeabor et al. | YES | YES |  |  |  |  | 5 | 100% |
| Osanyin et al., 2022 | YES | YES |  | 3 |  |  |  | 60% |
| Otu AA, et al., 2022 | YES | NO |  |  |  |  | 4 | 80% |
| Schmitz et al. | YES | YES |  |  | 3 |  |  | 60% |
| Shuaib et al | YES | YES | 4 |  |  |  |  | 80% |
| Tegegne et al | YES | YES |  |  |  | 5 |  | 100% |
| Tripathi et al. | YES | YES |  |  |  |  | 5 | 100% |
| Uba et al. | YES | YES |  |  | 4 |  |  | 80% |
| Udenigwe et al. | YES | YES | 5 |  |  |  |  | 100% |
| Udenigwe et al. | YES | YES | 5 |  |  |  |  | 100% |
