# Supplementary material for: Integrating digital health technologies into the healthcare system: Challenges and opportunities in Nigeria
Source: PLOS Digit Health. 2025 Jul 24;4(7):e0000928. doi: 10.1371/journal.pdig.0000928 (PMC12289021; doi:10.1371/journal.pdig.0000928)
Supplement: Attachment — Submitted filename: Response to Editors and Reviewer- DHT.docx [file pdig.0000928.s007.docx]

**Response to Editors**

**Manuscript Title:** Integrating Digital Health Technologies into the Healthcare System: Challenges and Opportunities in Nigeria
**Manuscript ID:** PDIG-D-24-00577

Dear Editors,

Thank you for your thoughtful feedback and the opportunity to revise our manuscript. We appreciate the time and effort invested by you and the reviewers in evaluating our submission. We have carefully considered all comments and have revised the manuscript accordingly to enhance its clarity, depth, and alignment with the journal’s expectations.

We have attached the following as part of our resubmission:

- A revised version of the manuscript (with tracked changes)
- A clean version of the revised manuscript
- A detailed point-by-point response to each reviewer’s comment (below)

We hope the revised version addresses all concerns and meets the publication standards of PLOS Digital Health.

Sincerely,
Dr. Ayodapo O. Jegede
On behalf of all co-authors

**Response to Reviewer #1**

We sincerely thank Reviewer #1 for the encouraging comments and insightful suggestions. Below, we respond to each point raised:

**Comment (Line 41):** The sentence implies a subset of 31 studies, while 31 was the total.
**Response:** Revised line 39 to read: “A total of 31 observational and experimental studies were included.” Line 41 has been removed for clarity.

**Comment (Line 85):** Typo – “holds” → “hold”
**Response:** Corrected as suggested.

**Comment (Lines 145–146):** Suggest including references to selected items discussed for broader context.
**Response:** We have added relevant references to enrich the background and theoretical grounding, enhancing the manuscript's value as a gateway to digital health scholarship.

**Comment (Line 270–271):** Clarify meaning of “non-literate” in context of SMS-based interventions.
**Response:** We have revised this section to clarify that "non-literate" refers to low health literacy rather than reading ability. This improves clarity regarding barriers to SMS intervention adoption.

**Table 3 Formatting Suggestions:**

1. Removed outer parentheses from all entries.
2. Added punctuation where necessary.
3. We appreciate the suggestion to split Table 3 into three separate tables. However, we have retained the single-table format intentionally to allow for integrated, side-by-side comparison of opportunities, challenges, and strategies across each theme.

**Section 276–304 (“Frequency of Occurrence of Themes”)**

- **Adjective use:** We reviewed and revised adjectives to better match reported frequencies.
- **Compact presentation:** The section now includes a compact summary table (Table 4), aligning with your suggestion.

**Lines 321, 346–347, 358, 401:** Language revised to remove self-promotion and allow the study’s merit to stand on its own. Line 401, the “Strengths of the Study” has been rephrased to “Key Findings”. Lines 402–409 have been removed, and the whole section rewritten as suggested.

**Line 389–390:**
The results in the “Characteristics of included studies” section have been expanded to illustrate the one-off nature and limited geographic scope of DHT implementations in Nigeria, with appropriate references to specific studies. Additionally, Table 2 has been updated to reflect these adjustments.

**Line 416–417:**
This content has been moved into the “Key Findings” section, consistent with your suggestion that it reflects a challenge rather than a methodological limitation.

**Response to Reviewer #2**

Thank you for your positive assessment and thoughtful comments. We appreciate your support of the manuscript’s focus and methodology. Below are our responses to your specific suggestions:

**Comment:** Include perspectives from local DHT product/service implementers and government agencies.
**Response:** We agree with this important observation. Our review was guided by the scope of available empirical studies at the time, most of which focused on user-level perspectives. As reflected in Table 2, 18 of the 31 studies (58.1%) involved healthcare workers, while 17 studies (54.8%) included patients or caregivers, and only 5 studies (16.1%) included community members or the general public. Only one study included policymakers, and none focused on private technology vendors or digital product implementers. We have clearly acknowledged this limitation in the Limitations section and emphasized in the Discussion the need for future research to actively engage local DHT developers, implementers, and government agencies.

**Comment (Line 99):** WHO recommendation reference missing.
**Response:** This has been corrected. The paragraph now reads:

"With a doctor-to-population ratio of approximately 1:9,083 [12], Nigeria falls well below the WHO’s recommended threshold of 2.3 skilled health professionals per 1,000 population [13]."

**Citation Added:**

World Health Organization. *Health workforce requirements for universal health coverage and the Sustainable Development Goals (Human Resources for Health Observer Series No. 17)*. Geneva: WHO; 2016. Available from: <https://iris.who.int/bitstream/handle/10665/250330/9789241511407-eng.pdf>

We are grateful for the reviewers' constructive feedback and hope the revised manuscript is now suitable for publication. Thank you once again for the opportunity to revise and resubmit our manuscript.
